# Effects of Surgical and Dietary Weight Loss Therapy for Obesity on Gut Microbiota Composition and Nutrient Absorption

**DOI:** 10.1155/2015/806248

**Published:** 2015-02-01

**Authors:** Antje Damms-Machado, Suparna Mitra, Asja E. Schollenberger, Klaus Michael Kramer, Tobias Meile, Alfred Königsrainer, Daniel H. Huson, Stephan C. Bischoff

**Affiliations:** ^1^Department of Nutritional Medicine, University of Hohenheim, Fruwirthstraße 12, 70599 Stuttgart, Germany; ^2^Singapore Centre on Environmental Life Sciences Engineering, Nanyang Technological University, Singapore 637551; ^3^Department of General, Visceral and Transplant Surgery, University of Tübingen, 72076 Tübingen, Germany; ^4^Department of General and Visceral Surgery, Chirurgische Klinik München-Bogenhausen, 81679 Munich, Germany; ^5^Center for Bioinformatics, University of Tübingen, 72076 Tübingen, Germany

## Abstract

Evidence suggests a correlation between the gut microbiota composition and weight loss caused by caloric restriction. Laparoscopic sleeve gastrectomy (LSG), a surgical intervention for obesity, is classified as predominantly restrictive procedure. In this study we investigated functional weight loss mechanisms with regard to gut microbial changes and energy harvest induced by LSG and a very low calorie diet in ten obese subjects (*n* = 5 per group) demonstrating identical weight loss during a follow-up period of six months. For gut microbiome analysis next generation sequencing was performed and faeces were analyzed for targeted metabolomics. The energy-reabsorbing potential of the gut microbiota decreased following LSG, indicated by the Bacteroidetes/Firmicutes ratio, but increased during diet. Changes in butyrate-producing bacterial species were responsible for the Firmicutes changes in both groups. No alteration of faecal butyrate was observed, but the microbial capacity for butyrate fermentation decreased following LSG and increased following dietetic intervention. LSG resulted in enhanced faecal excretion of nonesterified fatty acids and bile acids. LSG, but not dietetic restriction, improved the obesity-associated gut microbiota composition towards a lean microbiome phenotype. Moreover, LSG increased malabsorption due to loss in energy-rich faecal substrates and impairment of bile acid circulation. This trial is registered with ClinicalTrials.gov NCT01344525.

## 1. Introduction

Obesity is a worldwide health problem that reaches epidemic dimensions and is accompanied by a tremendously rising prevalence of related comorbidities, such as type 2 diabetes, hypertension, cardiovascular disease, nonalcoholic fatty liver disease, arthritis, depression, and certain cancers. In 2008, approximately 1.46 billion adults worldwide were overweight and 502 million adults were obese [1]. By now, mortality caused by overweight and obesity exceeds the number of deaths linked to underweight [2]. The success rates of suggested obesity prevention and treatment strategies including lifestyle modification, behavioural therapy, and pharmacotherapy are dissatisfactory and lack efficacy in the management of morbid obesity [3]. Surgical interventions are currently the most effective evidence-based approach towards clinically significant and sustainable weight loss along with reduction in mortality and obesity-related comorbidities [4, 5].

Bariatric operations experienced an exponential rise during the past few decades in matters of frequency, but also in the evolution of operation types and techniques [6–8]. Laparoscopic sleeve gastrectomy (LSG) is gaining popularity both as a single-staged and revisional operation and proved to be a simple and safe technique with promising short-term and midterm efficacy [9–11]. By partitioning and removal of a majority of the stomach parallel to downsizing the greater curvature while maintaining the gastrointestinal continuity, LSG was commonly classified as a solely restrictive procedure [12, 13]. The latest evidence recognizes a more sophisticated mechanism of LSG involving alteration of gut hormones such as ghrelin, peptide YY, and glucagon-like peptide [14]. Moreover, recent studies demonstrate an accelerated gastric emptying and small bowel transit time possibly also responsible for the weight-reducing effect [15–19]. Thus, LSG seems to manifest itself as metabolic operation by supporting the “hindgut theory” suggesting that an expedite delivery of unabsorbed, incompletely digested nutrients to the distal intestine results in stimulation of hormonal changes leading to improved glycemic control [20]. However, the complex mechanisms resulting in the beneficial effects of LSG and the impact of surgically modified food ingestion and digestion remain poorly understood.

Besides diet, lifestyle, genetics, and environment, the gut microbiota has been recognized most recently as a potential contributor to the mechanisms of obesity and the metabolic syndrome. The nutrient load is a key variable that can influence the human gut microbiota, which in turn plays a role in nutrient absorption and the regulation of nutrient harvest [21]. One of the main bacterial phyla, the Firmicutes, more efficiently extracts calories from carbohydrates than Bacteroidetes by fermentation of otherwise indigestible food components to short-chain fatty acids (SCFA). Such metabolites are absorbed through the intestinal mucosa [21, 22], thus maximizing the energy supply provided by the diet.

The bariatric procedure Roux-en-Y bypass has been shown to result in an increase in Firmicutes and decrease in Bacteroidetes in animal models [23] and humans [24, 25] and to reflect alterations due to the surgical induced anatomical changes in the gastrointestinal tract, while to the best of our knowledge no information is available on the impact of the increasingly popular, and, compared to gastric bypass, similarly effective bariatric sleeve gastrectomy.

In this context, we conducted a longitudinal pilot study over a period of six months including three time points, aiming to provide insight into the effect of LSG as well as a purely restrictive dietary approach for obesity therapy. We studied effects on gut microbiota, on the microbial metabolic capacity, as well as on faecal metabolic profiles using recent high-throughput “omics”-technologies. The solely restrictive and similar effective dietary therapy might enhance our understanding of other mechanisms than surgically induced food restriction that might be of importance for weight loss following LSG.

## 2. Methods

### 2.1. Weight Loss Interventions

Subject assignment to the dietary and surgical intervention was based on current evidence-based guidelines [26, 27].

#### 2.1.1. Laparoscopic Sleeve Gastrectomy (LSG)

LSG was performed at the University Hospital for General, Visceral and Transplant Surgery, Tübingen, Germany. Included patients met the criteria of the German national S3 guideline for the surgical treatment of morbid obesity [26]. The indication for LSG was approved by the local interdisciplinary case conference. LSG was performed by one surgeon (Klaus Michael Kramer) according to a standardized procedure as described previously [28]. Subjects undergoing LSG did not receive any kind of preoperative diet. Postoperative diet progression followed a gradual return to solid foods within a maximum of 8 weeks [29], with a caloric goal comparable to the group receiving the very low calorie diet (VLCD) (=800 kcal/d). A total energy intake around this magnitude three months after gastric resection is usually reported by our LSG patients to the dieticians guiding the post-operative nutrition care program. This is also confirmed by data indicating daily calorie intakes of 710 kcal at three months [30] and 930 ± 29 kcal/d at four months [31] postoperatively. Moreover, subjects were advised to follow a protein-rich, low-fat diet. In particular within the first three months the diet was very low in fiber, due to the postoperative nutrition care program ((1) liquids, (2) pureed, (3) soft solids, and (4) regular foods) as well as postoperative infeasibilities of fiber-rich, bulky foods.

#### 2.1.2. Very Low Calorie Diet Program (VLCD)

The multidisciplinary weight loss program (OPTIFAST 52) causes an average relative weight loss of 19.0% in men and 17.4% in women within one year [32]. Briefly, the program consists of a five-phase lifestyle modification over 52 weeks, including (i) a 1-week introduction during which a detailed nutrition analysis is performed; (ii) a 12-week period of very low calorie diet (VLCD) (800 kcal/day) during which participants consume a formula diet exclusively (daily consumption of 5 sachets at 160 kcal each, Optifast 800 formula, Nestlé Inc.); (iii) a 6-week refeeding phase; (iv) a 7-week stabilization phase; (v) a 26-week maintenance phase (the latter phase was not included for followup in this study). Besides medical examination, exercise units, and nutrition counselling all phases are accompanied by extensive behavioural modification lessons. More detailed information about the program is provided in a previous report [32].

### 2.2. Subject Selection and Biomaterial Sampling

Selected subjects were participants of a multicenter clinical trial, research project “Obesity and the Gastrointestinal Tract” within the Competence Network Obesity (registered with ClinicalTrials.gov NCT01344525), approved by the ethics committee of the University Hospital of Tübingen, Germany. Written informed consent was obtained from every subject prior to participation. Selection criteria included similar extent of weight loss, similar age, sex (only females were included), nondiabetics, and subject's affiliation to the same enterotype (Bacteroides-enterotype) to minimize interindividual variability. Faecal analysis was performed in 10 unrelated subjects with obesity grade III at three time points: one day before, as well as 3 and 6 months after LSG (*n* = 5) or the dietary weight loss regimen (*n* = 5), respectively. Short-chain fatty acids were analyzed in additional 10 subjects per group (*n* = 15 LSG, *n* = 15 VLCD), which met the same selection criteria. Results of gut microbiota analysis are shown for complete longitudinal datasets of 6 subjects (*n* = 3 LSG, *n* = 3 VLCD). None of the subjects had a history of chronic gastrointestinal problems or current gastrointestinal symptoms and none had received antibiotic and pre- or probiotic agents within 3 months before sample collection. Of the same passage, native faecal specimens were collected in stool collection tubes for metabolite extraction and also in tubes containing DNA stabilizer (Stratec Molecular, Berlin, Germany) for isolation of microbial DNA. All samples were stored at −80°C until analysis.

### 2.3. Analysis of Gut Microbiota by SOLiD Long-Mate-Paired Shotgun Sequencing

Faecal sample preparation and SOLiD (sequencing by oligonucleotide ligation and detection) shotgun sequencingwas performed as described previously [33]. Briefly, microbial DNA was isolated using the PSP Spin Stool DNA Plus Kit with lyses enhancer (Stratec Molecular, Berlin, Germany) as described by the manufacturer. Long-mate-paired libraries with mate-paired distances of 300–900 bp apart from the whole microbial DNA (with a maximum at 800 bp) were generated by randomly shearing in a microTube format using the Covaris S2 sonicator (Covaris, Woburn, MA, USA), according to the mate-paired library construction protocol (Applied Biosystems, Foster City, CA, USA). The fragmentation protocol was mildly adapted to duty cycle 5%, intensity 3, cycles per burst 200, at 4°C. Fragmentation times were adjusted to 15 sec. No size selection was performed. Finished libraries were clonally amplified on paramagnetic beads, deposited onto a glass slide, and sequenced according to standard Applied Biosystems protocols using the SOLiD 4 System (Applied Biosystems, Foster City, CA, USA).

### 2.4. Taxonomic, Functional, and Comparative Analysis of Sequencing Data

The procedure for data processing was performed as previously reported [33]. In brief, the first step of data processing involved conversion of reads to the “fasta” format, while all the reads with less than 20 base pairs length or with base quality values below 18 were discarded. Reads of length 40 bp or above from both forward and reverse files, together with their all mates (>=20 bp), were considered. The adapters from both the files (“T” from the forward mates and “G” from the reverse mates) were removed and all of these sequences were aligned against NCBI-NR database of nonredundant protein sequences [34] using BLASTX [35]. After performing the BLAST comparison, both output files were imported and analysed using the paired-end protocol of MEGAN 4 [36] (parameter settings* min score* = 35,* top percent* = 10, and* min support* = 25) using adjustments according to Mitra et al. [33]. Finally, all the files were compared based on their taxonomic content.

### 2.5. Metabolite Extraction from Faecal Samples

Specimens were homogenized with a spatula and a predetermined mass (1 g) was placed into test tubes using a faecal sample preparation kit (Roche Diagnostics GmbH, Mannheim, Germany). We added 10 mL of extraction buffer (EtOH (>96%)/10 mM phosphate buffer (pH 7,2), 85 : 15 (v/v)) that had been cooled on dry ice to each sample (1 : 10 [w/v]) and homogenized it for 2 min using a Vortex mixer. Extracts were then vigorously shaken on ice for 2.5 hours with an orbital shaker. Subsequently, specimens were sonicated at 70 W for 1 min in an ice bath using an ultrasonic homogenizer (Sonopuls HD 2070, Bandelin GmbH & Co. KG, Berlin, Germany). Next, faecal extracts were centrifuged (800 ×g, 0°C) for 10 min. The supernatant was separated and again centrifuged (20.800 ×g, 5°C, 10 min). The obtained supernatant was stored at −80°C until further analysis.

### 2.6. Mass Spectrometric Analysis of Faecal Fatty Acids and Bile Acids

A highly selective reversed phase LC-MS/MS analysis method performed in negative MRM detection mode was applied to determine the concentration of bile acids in faecal extracts. Samples were extracted via dried filter spot technique and measured by LC-ESI-MS/MS with an ABSciex 4000 QTrapTM tandem mass spectrometry instrument (AB Sciex, Darmstadt, Germany). Eicosanoids and other oxidized polyunsaturated fatty acids were determined by HPLC-tandem mass spectrometry (LC-MS/MS) with multiple reaction monitoring (MRM) in negative mode using a SCIEX API 4000 QTrap mass spectrometer with electrospray ionization instrument (AB Sciex, Darmstadt, Germany) [37].

For determination of faecal SCFA human stool samples were freeze-dried and subsequently analyzed using gas chromatography. Briefly, the samples were weighted; then an extraction solution (H_2_O, HCL 37%, 720 *μ*L heptanoic acid) and glass beads (*⌀*3 mm) were added and the mixture was homogenized for 5 min. After that it was left for 15 min and subsequently centrifuged for another 15 min (2.500 ×g at 4°C.) Concentrations of SCFA were determined in the supernatant, after adding trifluoroacetic acid, resting for 8 h and centrifugation for 90 min (2.500 ×g at 4°C), and addition of internal standards using a Shimadzu GC 14A gas chromatograph (Shimadzu, Kyoto, Japan) equipped with a capillary column Roti Cap-FFAP (30 m × 0,32 mm, df 0,5 *μ*m) (Carl Roth, Karlsruhe, Germany). Class-VP 4.2 software was used for data processing.

### 2.7. Multivariate Data Analysis and Statistics

Both taxonomically assigned sequencing reads and faecal metabolomics data were subjected to multivariate data analysis using the SIMCA-P+ 13.0 software (Umetric, Umea, Sweden). For the microbiome data, normalized Euclidean distances of the phylogenetic tree on each taxonomic level were calculated. For each dataset, a discriminant model was built with mean centred and unit variance scaled data using orthogonal partial least square discriminant analysis (OPLS-DA) to investigate differences between pre- and postintervention effects. In the model, the postintervention classification conjoined month 3 and month 6 data aiming to identify time-persistent markers. The quality and robustness of the models were evaluated by the cumulative *R* ^2^ *Y* and *Q* ^2^ values, where *R* ^2^ *Y* is a quantitative measure of the goodness of fit and *Q* ^2^ summarizes the model's goodness of prediction. 7-fold cross validation and permutation tests based on 200 permutations were performed to test for validity of the model and overfit of class assignment. Identification of prominent differences between pre- and postintervention was performed according to the method suggested by Rantalainen et al. [38]. In brief, the predictive regression coefficients (*P*(corr)) from each model were used to identify the most prominently changing variables, considering a* P*(corr) > 0.75 and* P*(corr) < −0.75, respectively, as cutoff for significance (∗). More detailed analysis of parameters selected by multivariate analysis was performed using conventional statistical tests. Accordingly paired *t*-tests or Wilcoxon matched pair tests were used for comparison of two time points and repeated measures analysis of variance (one-way-ANOVA with Tukey post hoc testing) or Friedmann test for time courses were conducted after testing for Gaussian distribution using D'Agostino-Pearson tests. Absolute and percentage values are presented as mean ± SEM.

## 3. Results

### 3.1. Weight Loss and Clinical Parameters

The 10 obese individuals were all female, age 48 ± 3 years, and nonsmokers. Baseline anthropometric measures weight, BMI, and waist circumference did not significantly differ between both intervention groups. Bariatric sleeve operation resulted in a relative weight loss (RWL) of 16.1 ± 1.1% after 3 months and 23.9 ± 1.6% at months 6. There were no significantly different weight loss characteristics (absolute and relative weight loss) after dietary intervention, which resulted in a RWL of 17.2 ± 0.8% at month 3 and 24.6 ± 0.8% after 6 months of intervention. The detailed pre- and postoperative anthropometric and clinical parameters are given in Table 1.

### 3.2. Gut Microbiota Composition

**Figure 1:**
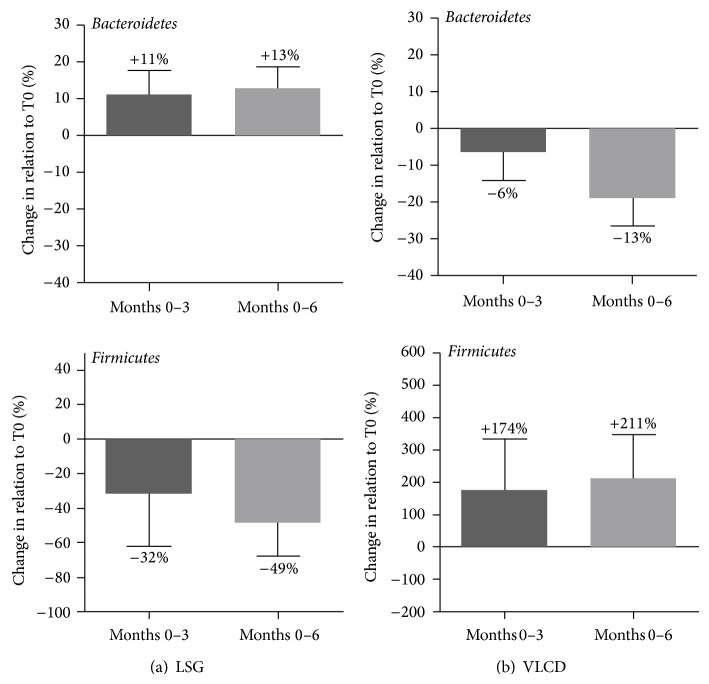
Alterations in the main phyla Bacteroidetes and Firmicutes of the human intestinal microbiota after three and six months of weight loss therapy for morbid obesity: (a) laparoscopic sleeve gastrectomy (LSG) and (b) very low calorie diet (VLCD).

**Figure 2:**
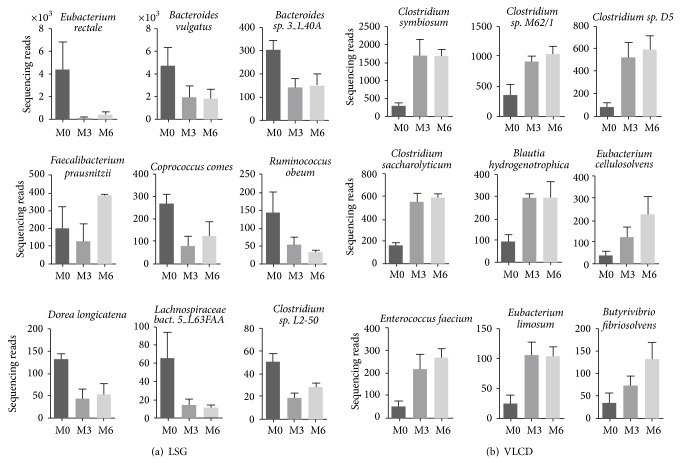
Human gut microbiota changes on the taxonomic species level induced by weight loss therapy for morbid obesity: (a) laparoscopic sleeve gastrectomy (LSG) and (b) very low calorie diet (VLCD). Shown are all bacterial species for which changes were significantly defined as a* P*(corr) > 0.75 or <−0.75 derived from OPLS-DA models.

**Figure 3:**
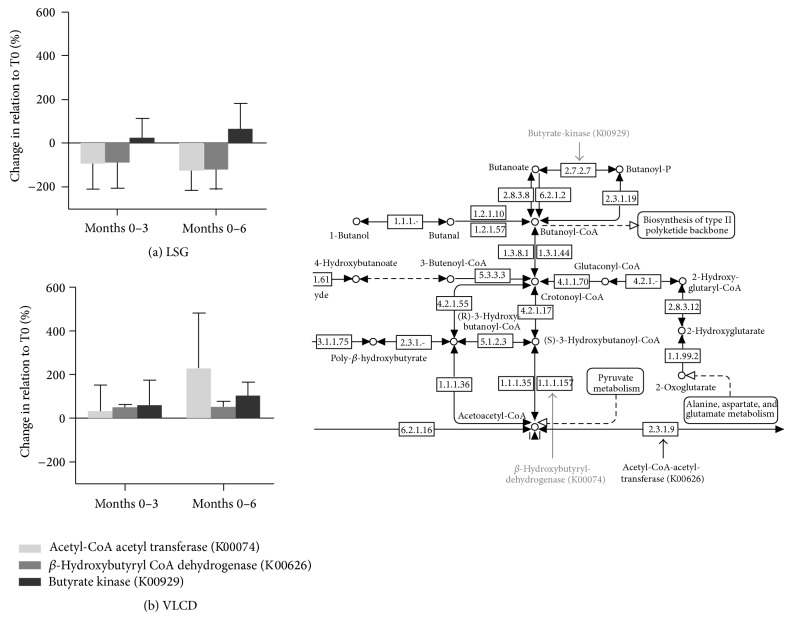
KEGG orthologues representing the microbial metabolic capacity of the human gut microbiota for butyrate fermentation after weight loss therapy for morbid obesity: (a) laparoscopic sleeve gastrectomy (LSG) and (b) very low calorie diet (VLCD). Shown are three KEGG orthologues (K00074, K00929, and K00626) with key functions in the fermentation pathway denoted in the KEGG map extract on the right bottom corner.

Analysis of similarities between subjects and time points based on calculation of normalized Euclidean distances revealed profound changes of faecal microbial community composition after LSG, whereas the VLCD intervention resulted in a more varied picture with less distinct dissimilarity between time points (see SI-Figure  1 in Supplementary Material available online at http://dx.doi.org/10.1155/2014/806248). Detailed post-interventional alterations of bacterial groups on all taxonomic levels were identified using coefficient plots of OPLS-DA models (SI-Figure  2 and SI-Table  1). Both interventions resulted in changes of the Bacteroidetes : Firmicutes ratio, but with an inverse relationship between the main phyla (Figure 1). While LSG resulted in a distinct increase in Bacteroidetes (*P*(corr) = 0.85) and a decline in the abundance of Firmicutes (*P*(corr) = −0.85), the dietary intervention resulted in reduced Bacteroidetes in favour of Firmicutes. The Bacteroidetes/Firmicutes ratio changed from 14.2 ± 7.9 to 4.8 ± 0.5 (month 3) and 3.7 ± 1.4 (month 6) following dietary intervention, whereas in the LSG group, this ratio increased from 5.9 ± 2.1 to 10.4 ± 1.4 (month 3) and 13.8 ± 3.0 (month 6). In the LSG group, the number of Bacteroidetes showed a negative correlation with body weight (*r* = −0.61, *P* = 0.05) while Firmicutes numbers were positively correlated with body weight (*r* = 0.65, *P* = 0.05). In contrast, in the dietary group body weight correlated significantly positive with Bacteroidetes (*r* = 0.67, *P* = 0.05) and inversely with Firmicutes (*r* = −0.68, *P* < 0.05). A figure of the abundance of bacterial groups at the class level for each subject and timepoint is presented in SI-Figure  3.

In each group 9 bacterial species of the gut microbiota were identified, which consistently altered pre- to postintervention.  Figure 2 depicts changes on species level for both groups. Apart from two species of the Bacteroidetes genus in the LSG group (Bacteroides* sp. 3_1_40A P*(corr) = −0.92 and* Bacteroides vulgatus P*(corr) = −0.78), all alterations occurred in species assigned to Firmicutes phylum. Except for a postoperative increase in* Faecalibacterium prausnitzii* (*P*(corr) = 0.81), LSG resulted in a decrease of several Firmicutes species. In the latter group all Bacteroidetes subgroups increased (*P*(corr) = 0.87). The postoperative reduction in Firmicutes following LSG was caused by a decline of bacterial subgroups belonging to Clostridial clusters IV and XIV, which include species of* Clostridium*,* Eubacterium*,* Faecalibacterium*,* Dorea*, and* Coprococcus*. In particular known butyrate-producing bacteria (*Coprococcus sp. P*(corr) = −0.88,* Eubacterium rectale P*(corr) = −0.79*, Ruminococcus obeum P*(corr) = −0.82, and* Lachnospiraceae bacterium 5_1_63FFA P*(corr) = −0.85) experienced a decline in the postoperative period. In contrast, we observed a rise in specific Firmicutes species in the course of the VLCD program. These changes also pertained to butyrate-producing bacterial strains (*Butyrivibrio fibrisolvens P*(corr) = 0.80*, Clostridium saccharolyticum* (*P*(corr) = 0.94),* Eubacterium limosum* (*P*(corr) = 0.90), and* Blautia hydrogenotrophica* (*P*(corr) = 0.88)), but here resulting in an increase in those microbes.

### 3.3. Microbial Metabolic Capacity for Butyrate Fermentation Pathway

Based on the findings of predominantly alterations in butyrate-synthesizing bacteria, we analyzed the microbial metabolic capacity of the gut microbiota for the main KEGG orthologues (acetyl-CoA-acetyl transferase (K00074), butyrate kinase (K00929), and beta-hydroxy-butyryl-CoA dehydrogenase (K00626)) involved in the fermentation pathway to butyrate. Summed values tended to result in a mean increase of 380.2 ± 52.5% in the metabolic capacity of these KEGG orthologues for butyrate fermentation after 6 months of VLCD program (K00074 227.3 ± 254.2%, K00929 102.3 ± 61.5%, and K00626 50.5 ± 27.45%) and a decrease for the LSG intervention −184.4 ± 63.0% (K00074 −126.7 ± 89.7%, K00929 64.4 ± 118.0%, and K00626 −122.2 ± 91.0%), respectively (Figure 3).

### 3.4. Faecal Fatty Acid Excretion and Bile Acid Metabolism

Both weight loss interventions, the VLCD regimen and LSG, did not reveal significant differences in faecal butyrate content and other SCFA, possibly because of low prebiotic substrate levels in the diet. Data on faecal SCFA concentrations were also performed in 10 additional and similarly matched subjects per intervention group and confirmed unaltered SCFA concentrations after both weight loss strategies (Table 2).

**Figure 4:**
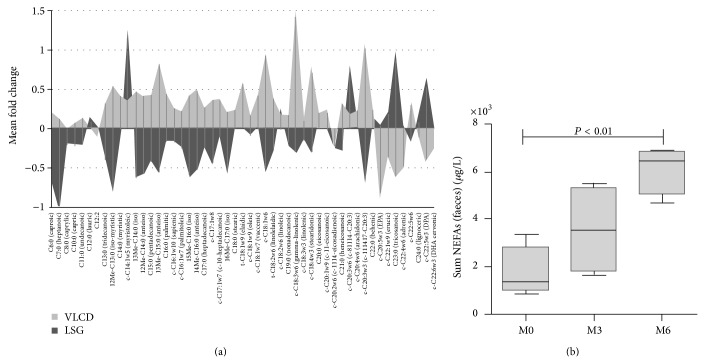
Faecal excretion of middle- and long chain nonesterified fatty acids (NEFAs): (a) mean fold changes of NEFA excretion 6 months after weight loss therapy for morbid obesity (light grey: laparoscopic sleeve gastrectomy (LSG) and dark grey: very low calorie diet (VLCD)). (b) Summed faecal concentrations of NEFAs in the course after laparoscopic sleeve gastrectomy (LSG).

Analysis of faecal excretion of a total of 51 middle- and long-chain nonesterified fatty acids (NEFAs) revealed a trend to a decline in faecal concentrations of 31 NEFAs 6 months after the VLCD program (ns (*P* = 0.2283) for summed values); contrariwise, there was an increased loss of overall 40 different fatty acids 6 months after LSG compared to preoperative values resulting in a faecal excretion of summed fatty acid concentrations from preoperative 1,726 ± 1,093 *μ*M to 3,554 ± 1,872 *μ*M at month 3 and 6,106 ± 1,005 *μ*M at month 6 (*P* = 0.005) (Figure 4).

**Figure 5:**
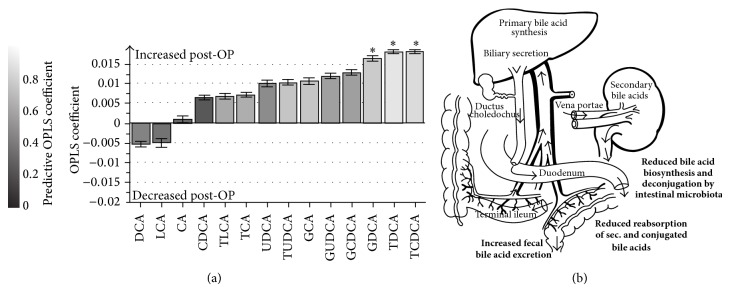
Faecal bile acids alterations six months after laparoscopic sleeve gastrectomy (LSG). OPLS-DA coefficient plot showing the increase or decrease in concentration for each of the bile acids identified by targeted profiling. The model compares 6 months post-LSG with preoperative values. The coefficient along the *y*-axis is a measure of both the magnitude and direction of change of bile acid concentrations. ∗ indicates which metabolites contribute significantly to class discrimination (pre- to post-LSG;* P*(corr) > 0.75 and* P*(corr) < −0.75) corresponding to the color scale code shown on the left hand. Error bars derived from calculation of Jack-knifing uncertainty measures. (b) Suggested impact of LSG on extrahepatic bile acid circulation based on the presented data.

Faecal metabolomics analysis of bile acids after LSG indicates postoperative increases in conjugated bile acids, while on contrary secondary bile acids (lithocholic acid, LCA; deoxycholic acid, DCA) slightly tended to decrease (Figure 5), while there was no change in the VLCD group (data not shown). The conjugated bile acids, glycodeoxycholic acid (GDCA,* P*(corr) = 0.76), taurodeoxycholic acid (TDCA,* P*(corr) = 0.93), and taurochenodeoxycholic acid (TCDCA,* P*(corr) = 0.84), most prominently contributed to separation of pre- to postoperative profiles. This was also linked to a certain decrease of the microbial capacity for primary and secondary bile acid biosynthesis, which was averagely reduced by −23.8 ± 33.3% at month 3 and −34.2 ± 23.9% at month 6 compared to pre-LSG (SI-Figure 4). Correlation analysis of gut microbial changes observed after LSG also showed positive associations between faecal secondary bile acid concentrations and postoperatively reduced microbial species (correlation of LCA with* Faecalibacterium prausnitzii*: *r* = 0.68, *P* = 0.04; DCA with* Clostridium sp. L2-50*: *r* = 0.73, *P* = 0.03;* Coprococcus comes*: *r* = 0.63, *P* = 0.04; and* Lachnospiraceae bacterium: r* = 0.81, *P* = 0.01, resp.), though conjugated bile acids indicated negative correlation coefficients (SI-Figure 5).

## 4. Discussion

In this study we analyzed longitudinal data in the course of two different approaches of predominantly restrictive weight loss therapies, the bariatric procedure LSG, and a standardized calorie restricted formula diet.

The bariatric procedure LSG was followed by a clear reduction of the Firmicutes/Bacteroidetes ratio. The change in main phyla was also observed in studies, in which subjects reduced body weight with caloric restriction and physical activity [39], fat- or carbohydrate-reduced diets [40], and Roux-en-Y gastric bypass (RYGB) [25]. Thus, one driving factor of the altered faecal microbiota composition might be the bacterial adaptation to caloric restriction. Due to the associated fermentation activity, occurrence of lower levels of Firmicutes following LSG could result in a reduction of energy harvest besides caloric restriction and thus might benefit sustained weight loss and maintenance. However, this was not clearly confirmed by our data on a functional level as shown by the unaltered faecal SCFA concentrations. Possibly, the expected decrease in SCFA production would become visible only if subjects would be challenged at the time of faecal analysis to substantial amounts of prebiotics and fibres, the substrates needed by the bacteria to generate SCFA. On the other hand, we observed a decline in butyrate-producing bacterial species after the surgical procedure. These modifications indeed point to a postoperatively reduced dietary intake of complex carbohydrates with prebiotic properties, especially resistant starch [22], which is consistent with the nutrition care program with slow progression from liquids to regular foods [29] as well as postoperative infeasibilities of fiber-rich, bulky foods after stomach resection. This change in diet after LSG can be explained by the anatomically induced food restriction, as well as a suggested more pronounced feeling of satiety induced by hormonal modulation [41]. Hence, in our subjects the observed modulation of the intestinal microbiota following LSG operation could be attributed to dietary influences, like reduced caloric restriction and fiber consumption.

Our data illustrate the less “invasive” impact of LSG, not only regarding anatomy but also regarding the gut microbiota, compared to RYGB. The latter procedure has been shown to lead to profound alterations of the intestinal microbiota [23–25]. Changes after RYGB can be explained to a large extent by an increased acid exposure to the gastric remnant and shortened small intestine as well as input of dissolved oxygen in the small bowel favouring growths of facultative anaerobe bacteria [24]. Moreover, the typical small intestinal microbiota might be relocated to the large intestine due to the more rapid flux of incompletely digested nutrients into the proximal gut [24]. These observations may contribute to creation of a cytotoxic environment in the bowel, [42] also leading to shifts in the gut microbial-host metabolic crosstalk as demonstrated by faecal, urinary, and host metabolomic profiles [23, 42, 43]. Thus, by maintaining the gastrointestinal continuity, LSG causes only moderate microbiota alterations with less severe negative consequences. Moreover, we also observed beneficial microbial changes during weight loss induced by LSG.* Eubacterium rectale*, which decreased after LSG operation, has previously been shown to positively correlate with obesity-related comorbidities [44], thus pointing to an influence of this microbe on host metabolic status. Furthermore, the study of Furet et al. [25], suggests that* Faecalibacterium prausnitzii* plays a role in low-grade inflammation pathologies such as obesity and diabetes and reports a reduction of this bacterial species after RYGB. Here we present that also LSG resulted in a decrease in* Faecalibacterium prausnitzii* numbers in obese subjects with preoperative impaired glucose tolerance.

In contrast, a dietary intervention with VLCD followed by a progressive adaptation to a low-fat, high-fiber, and energy-restricted diet resulted in a reduction of the Bacteroidetes/Firmicutes ratio along with weight loss. Uniformly, increased bacterial strains exclusively appertained to Firmicutes. Particularly,* Butyrivibrio fibrisolvens*,* Clostridium saccharolyticum*,* Eubacterium limosum,* and* Blautia hydrogenotrophica* represent bacteria, whose capability is the synthesis of the SCFA butyrate through fermentation of nondigestible nutrients in the upper GI tract. Moreover, growth of* Coprobacillus* and* Holdemania*, as well as* Eubacterium cellulosolvens* and* Clostridium saccharolyticum,* is favoured by an increase in fiber intake. Reasons for the modulation of microbiota composition might be the initial supply of the soluble fiber inulin included in the formula diet and the subsequent increased intake in dietary fiber, which was introduced by intensive nutritional training. Acetate and propionate, which are absorbed and transported to liver and other peripheral organs serving as substrates for gluconeogenesis and lipogenesis, represent an additional energy source for the host. In contrast, butyrate is primarily an energy substrate for colonic epithelial cells with positive properties in regard to integrity of the intestinal barrier [45]. Thus, despite an overall increase in Firmicutes at the phylum level following VLCD, which is generally associated with increased energy harvest for the host, the specific growth of the Firmicutes-related butyrate-producing species may result in positive metabolic effects. Nondigestible carbohydrates/prebiotics have been shown to reduce food intake by modulating the production of gastrointestinal peptides [46, 47]. Moreover, studies in murine models showed that activation of intestinal GLP-1 cells improves glycemic and insulin response [47]. This is accompanied by an improved expression and activity of proteins, which are involved in the intestinal barrier function and additionally reinforced by normalization of the intestinal endocannabinoid system response (reviewed in [48]). The increase in beneficial butyrate-producing microbes induced by soluble fiber supplementation might propose a possibility to countervail the observed postoperative declines in these microbial species following LSG.

The functional analysis of the metabolic capacity suggests an increased expression of enzymes, which are involved in butyrate fermentation, and thus an increased enzymatic potential for fermentation of acetyl CoA to butyrate after the VLCD program and vice versa for LSG. However, the observed gut microbial alterations in both intervention groups did not support any consequences for energy harvest as indicated by unaltered faecal SCFAs. This may be again due to a too limited substrate supply, such as fiber and resistant starch during both restrictive therapies to provoke changes on a functional level.

Favorable changes in the blood lipid profile with dietary restriction and weight loss were only induced by VLCD. The postoperative increases of total cholesterol and LDL 6 months after LSG may be explained by consumption of foods with unfavorable fatty acid patterns, especially after adaptation to the resected stomach (> three months postoperatively), and due to the lack of a guided nutrition counseling/lifestyle program for these subjects, as it was performed in the VLCD group. The guided group-based physical exercise classes in the VLCD group may also have induced the increase in HDL concentrations, which were not observed in the LSG patients.

Interestingly, we found increased faecal concentrations of middle- and long-chain fatty acids following LSG, whereas VLCD intervention resulted in lower concentrations compared to baseline. Dietary fat intake usually correlates with faecal fat excretion [49], which was in line with a decline in faecal fatty acid loss in the VLCD group after a period of food restriction, especially a reduced dietary fat consumption. The reciprocal finding after LSG points to a postoperative state of (moderate) malabsorption with an augmented faecal excretion of fatty acids. In recent years, evidence was raising that resection of the stomach leads to an accelerated gastric emptying by increased peristalsis [15–18]. Thus, the resulting delivery of unabsorbed, incompletely digested nutrients to distal intestinal areas might result in an increased loss of energy via faeces. Augmented faecal fat excretion is also observed after RYGB [50, 51], pointing to equal mechanisms of LSG and RYGB with regard to some intestinal malabsorption as a mechanism for weight loss that can be explained by accelerated gastric emptying and small bowel transit time. This subject should be addressed in further investigations analyzing total caloric contents in faeces before and after LSG to evaluate its impact on caloric load and absorption.

The faecal bile acids data further indicate a surgically induced alteration in the enterohepatic circulation. This is in line with the observation of increased faecal excretion of middle- and long-chain fatty acids, as bile acids play an important role in fat digestion and absorption. The primary bile acids undergo bacterial degradation in the intestine, whereat the secondary bile acids DCA, LCA, and their oxidation products are produced. The data suggest a reduced metabolic capacity by the postoperative intestinal microbiota for this process. Salts of conjugated bile acids are effective solvents, which provide absorption of lipids in the intestine. These lipids are almost completely absorbed and revert to liver and gall bladder via enterohepatic circulation. Besides a possible influence of gut microbial alterations, the fact that the faecal excretion of conjugated bile acids increased postoperatively may also be explained by a faster gastrointestinal passage possibly leading to a certain level of bile acid malabsorption.

A limitation of the study is the fact that the postoperative diet was not exactly matched to the VLCD intervention, which impedes a reliable analysis to dissect whether the observed changes were due to the LSG anatomical changes or to dietary restriction after surgery. Due to the small number of patients subjected to the sequencing analysis of the intestinal microbiome, this study has to be recognized as a pilot study, which needs to be confirmed in further studies. However, we focused on a time series in well-matched subjects for identification of time-persistent alterations using information from detailed deep sequencing techniques. In contrast to Zhang et al. [24], who compared three obese patients who underwent bariatric gastric bypass operation with three unrelated obese subjects without intervention, the approach presented here aimed at identification of intraindividual intervention-induced changes. Moreover, a strength of our study is that, in contrast to other published studies analysing gut microbiota composition in obesity, our focus was not only on a description of a shift of entire bacterial communities like the main phyla but also on detailed analysis of gut microbiota changes down to the species level.

## 5. Conclusions

By preserving the gastrointestinal continuity LSG resulted only in moderate alterations of the intestinal microbiota. The modulations can be explained by weight loss and dietary food restriction, most likely by reduced fiber consumption, as shown by the increase in Bacteroidetes/Firmicutes ratio and decline in butyrate-producing bacterial strains. In contrast, VLCD leads to an increase in butyrate-producing bacteria, which can be explained by soluble fiber supply, pointing to a possibility for supplementation with favourable effects on gut microbiota also in the postoperative period after LSG. Moreover, our data indicate a state of moderate malabsorption with loss of energy-rich faecal substrates and altered bile acid metabolism induced by LSG, which question its classification as solely restrictive procedure.

## Supplementary Material

SI-Figure 1. Dissimilarity- and cluster analysis of the phylogenetic trees in the course of weight loss therapy for obesity derived from sequencing of the intestinal microbiome.
SI-Figure 2. OPLS-DA coefficient plots for identification of pre-to post-interventional gut microbial changes on all taxonomoic levels during Laparoscopic sleeve gastrectomy and Very low calorie diet.
SI-Figure 3. Abundance of bacterial groups at the class level in 6 obese subjects undergoing weight loss therapy obtained by SOLiD shotgun sequencing during a period of 6 months.
SI-Figure 4. KEGG analysis of the microbial metabolic capacity for primary and secondary bile acid synthesis after intervention with laparoscopic sleeve gastrectomy.
SI-Figure 5. Correlation and cluster analysis of fecal bile acids with intestinal microbes, which changed following laparoscopic sleeve gastrectomy.
SI-Table 1. Alterations of the intestinal microbiota on different taxonomic levels following a) laparoscopic sleeve gastrectomy and b) Very low calorie diet-induced weight loss in obese subjects.

## Figures and Tables

**Table 1:** Anthropometric and clinical parameters in the dietary (VLCD) and sleeve gastrectomy (LSG) intervention group before (month 0) intervention and after 3 and 6 months.

	VLCD				LSG			
	Month 0	Month 3	Month 6	*P* ^*^ trend	Month 0	Month 3	Month 6	*P* ^*^ trend
Weight [kg]	121.1 ± 4.9	100.4 ± 4.5	91.3 ± 3.9	*0.0014* ^**^	127.5 ± 1.2	107.0 ± 2.4	97.1 ± 3.0	*<0.0001* ^***^
BMI [kg/m^2^]	40.2 ± 1.0	33.2 ± 1.0	30.3 ± 0.6	*<0.0001* ^***^	45.8 ± 0.9	38.4 ± 1.2	35.0 ± 1.3	*<0.0001* ^***^
Waist [cm]	114.7 ± 5.2	104.8 ± 1.9	99.3 ± 1.7	*0.0212* ^*^	138.3 ± 3.6	122.0 ± 4.7	112.0 ± 4.2	*0.0027* ^**^
Weight loss [kg]		20.7 ± 0.4	29.8 ± 1.0	*<0.0001* ^***^		20.5 ± 1.2	30.4 ± 1.8	*0.0017* ^**^
RWL [%]		17.2 ± 0.8	24.6 ± 0.8	*0.0002* ^***^		16.1 ± 1.1	23.9 ± 1.6	*0.0044* ^**^

BP syst. [mmHG]	120.0 ± 4.5	108.3 ± 7.9	108.3 ± 1.3	*0.2352 *	143.3 ± 13.7	110.0 ± 8.9	106.7 ± 2.6	*0.0351* ^*^
BP diast. [mmHG]	83.3 ± 1.3	75.0 ± 3.9	73.3 ± 1.3	*0.0321* ^*^	85.0 ± 9.7	66.7 ± 2.6	70.0 ± 0.0	*0.1002 *
Glucose [mg/dL]	102.0 ± 2.1	96.0 ± 0.8	88.3 ± 2.5	*0.0010* ^***^	125.3 ± 10.4	94.3 ± 7.6	105.0 ± 3.8	*0.0436* ^*^
HbA1C [%]	5.6 ± 0.1	5.3 ± 0.2	5.3 ± 0.0	*0.2186 *	5.5 ± 0.1	5.3 ± 0.1	5.3 ± 0.2	*0.5705 *
Triglycerides [mg/dL]	218.0 ± 30.9	113.7 ± 6.1	125.7 ± 9.1	*0.0039* ^**^	169.3 ± 17.4	119.0 ± 7.5	117.0 ± 1.6	*0.0086* ^**^
Cholesterol [mg/dL]	224.7 ± 15.9	164.0 ± 2.4	176.3 ± 16.3	*0.0165* ^*^	206.0 ± 17.5	188.3 ± 16.1	218.7 ± 16.9	*0.4640 *
HDL [mg/dL]	51.3 ± 2.7	42.3 ± 1.8	54.3 ± 2.9	*0.0147* ^*^	52.0 ± 3.9	44.7 ± 4.2	48.3 ± 4.3	*0.4827 *
LDL [mg/dL]	143.3 ± 12.8	104.3 ± 4.5	108.7 ± 11.5	*0.0383* ^*^	133.0 ± 21.2	118.3 ± 21.4	143.0 ± 20.8	*0.7147 *

^*^Within group comparison.

**Table 2:** Fecal short-chain fatty acid concentrations (mmol/100 g dry weight) in the dietary (VLCD; *n* = 15) and sleeve gastrectomy (LSG, *n* = 15) intervention groups before intervention (month 0) and after 3 and 6 months.

	VLCD				LSG			
	Month 0	Month 3	Month 6	*P* trend	Month 0	Month 3	Month 6	*P* trend
Acetate	20.3 ± 3.8	15.6 ± 2.5	29.6 ± 1.9	*0.4041 *	10.3 ± 1.7	16.5 ± 4.3	14.2 ± 3.0	*0.3909 *
Propionate	5.8 ± 1.2	3.5 ± 0.5	3.7 ± 0.5	*0.0954 *	3.5 ± 0.7	3.7 ± 0.8	3.5 ± 0.7	*0.9743 *
n-Butyrate	4.5 ± 1.0	2.5 ± 0.6	2.5 ± 0.5	*0.1095 *	2.2 ± 0.4	2.2 ± 0.6	2.5 ± 1.5	*0.9232 *
i-Butyrate	0.6 ± 0.1	0.5 ± 0.1	0.5 ± 0.1	*0.3393 *	0.4 ± 0.1	0.4 ± 0.1	1.0 ± 0.4	*0.1973 *
i-Valeriate	0.8 ± 0.1	0.7 ± 0.1	0.7 ± 0.1	*0.8427 *	0.6 ± 0.1	0.6 ± 0.1	0.6 ± 0.1	*0.8458 *
Caproate	0.2 ± 0.0	0.2 ± 0.0	0.2 ± 0.0	*0.6400 *	0.2 ± 0.0	0.2 ± 0.0	0.2 ± 0.0	*0.8547 *
Total SCFA	30.7 ± 5.9	22.4 ± 3.8	21.9 ± 2.7	*0.2862 *	18.2 ± 2.6	23.7 ± 6.3	24.9 ± 5.3	*0.6192 *

SCFA: short chain fatty acids.
