# Supplementary material for: Effects of Surgical and Dietary Weight Loss Therapy for Obesity on Gut Microbiota Composition and Nutrient Absorption
Source: Biomed Res Int. 2015 Feb 1;2015:806248. doi: 10.1155/2015/806248 (PMC4330959; doi:10.1155/2015/806248)
Supplement: Supplementary file 1 — SI-Figure 1. Dissimilarity- and cluster analysis of the phylogenetic trees in the course of weight loss therapy for obesity derived from sequencing of the intestinal microbiome. SI-Figure 2. OPLS-DA coefficient plots for identification of pre-to post-interventional gut microbial changes on all taxonomoic levels during Laparoscopic sleeve gastrectomy and Very low calorie diet. SI-Figure 3. Abundance of bacterial groups at the class level in 6 obese subjects undergoing weight loss therapy obtained by SOLiD shotgun sequencing during a period of 6 months. SI-Figure 4. KEGG analysis of the microbial metabolic capacity for primary and secondary bile acid synthesis after intervention with laparoscopic sleeve gastrectomy. SI-Figure 5. Correlation and cluster analysis of fecal bile acids with intestinal microbes, which changed following laparoscopic sleeve gastrectomy. SI-Table 1. Alterations of the intestinal microbiota on different taxonomic levels following a) laparoscopic sleeve gastrectomy and b) Very low calorie diet-induced weight loss in obese subjects. [file 806248.f1.pdf]

Supporting information 1: Figures

SI-Figure 1: Dissimilarity- and cluster analysis of the phylogenetic trees in the course of weight loss therapy for obesity derived from sequencing of the intestinal microbiome. *(A) (red): Laparoscopic sleeve gastrectomy (subject numbers LSG 1-3), (B) (green): Very low calorie diet (subject numbers VLCD 1-3). The color code of the heatmap corresponds to values of the normalized Euclidian distances of the phylogenetic trees.*

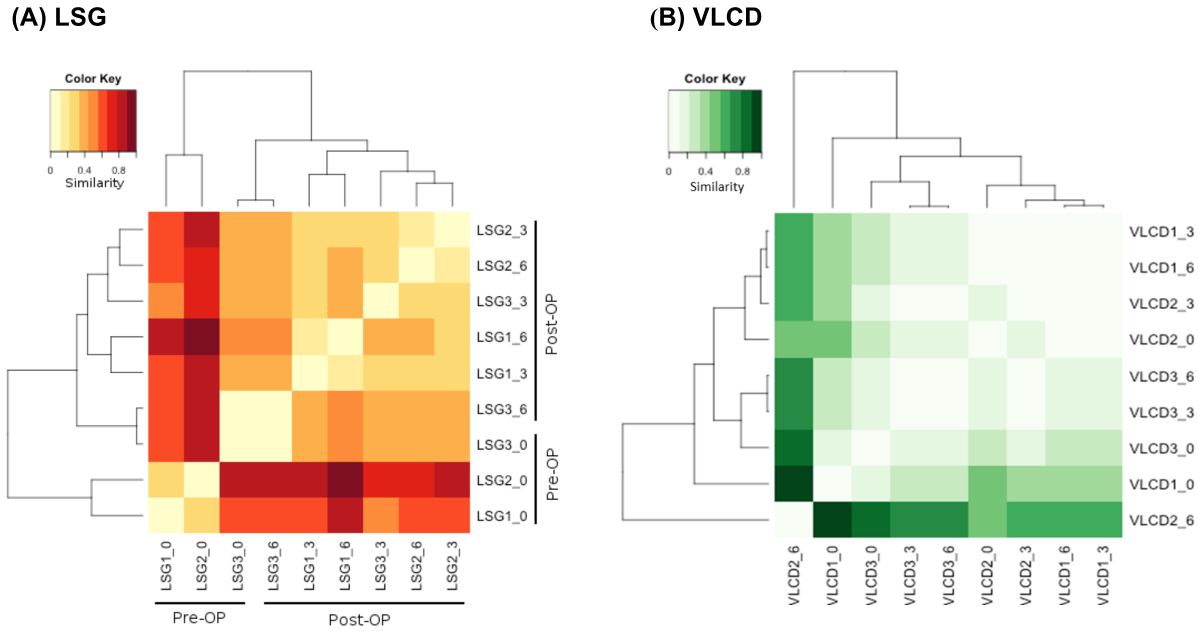

SI-Figure 2: OPLS-DA coefficient plots for identification of pre-to post-interventional gut microbial changes on all taxonomoic levels during *Laparoscopic sleeve gastrectomy (LSG; SDC-Fig. 2a)* and *Very low calorie diet (VLCD; SDC-Fig. 2b). Labeled are bacterial groups with predictive OPLS coefficient p(corr)>0.75 and < -0.75, respectively, as indicated by the color code shown on the left.*

Fig. 2a) LSG

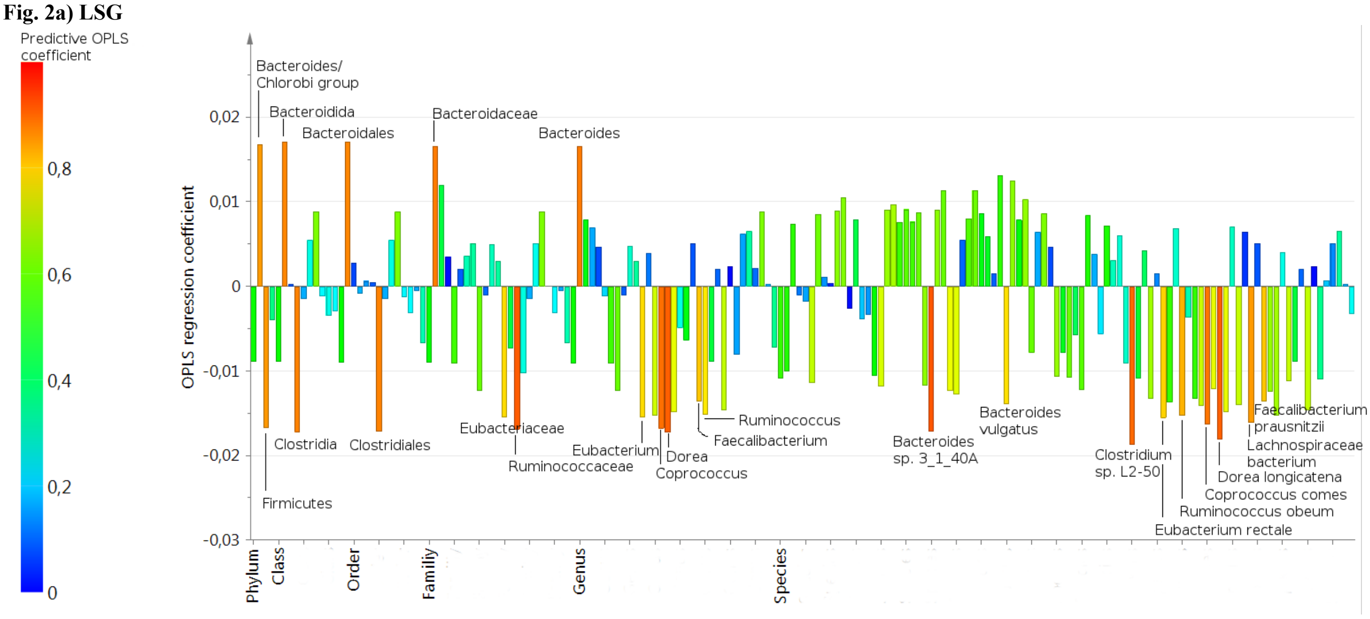

Model statistics: Q^2=0.337, R^2X=0.265

Fig. 2b) VLCD

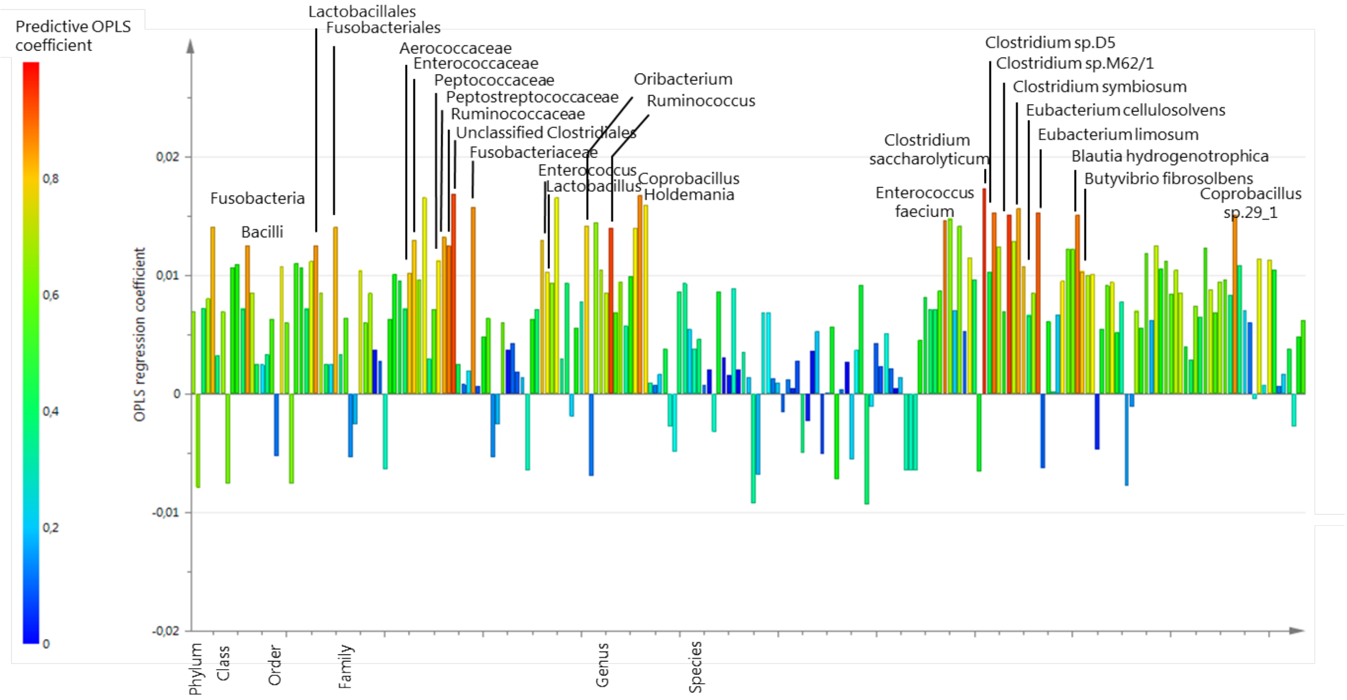

Model statistics: Q^2=0.56, R^2X=0.216

SI-Figure 3: Abundance of bacterial groups at the class level in 6 obese subjects undergoing weight loss therapy obtained by next-generation sequencing (SOLiD shotgun sequencing) during a period of 6 months (timepoints 0, 3, and 6 months): *(A) Laparoscopic sleeve gastrectomy (subject numbers: LSG 1-3) and (B) Very low calorie diet (subject numbers: VLCD 1-3).*

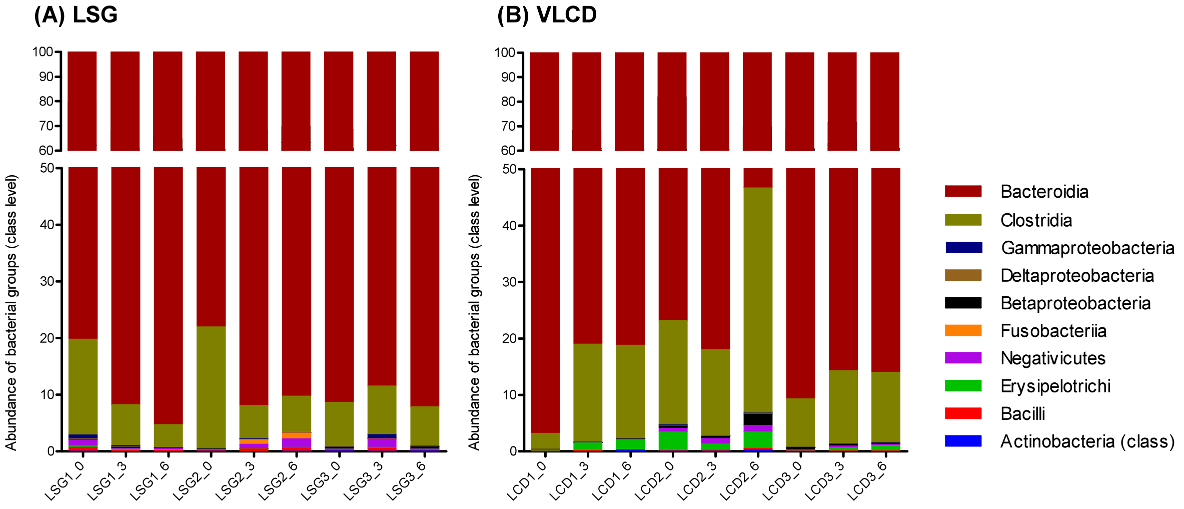

SI-Figure 4: KEGG analysis of the microbial metabolic capacity for primary and secondary bile acid synthesis after intervention with **laparoscopic sleeve gastrectomy** *(subject numbers LSG 1-3, timepoints 0, 3, and 6 months). Only a section of the biosynthesis pathway is shown.*

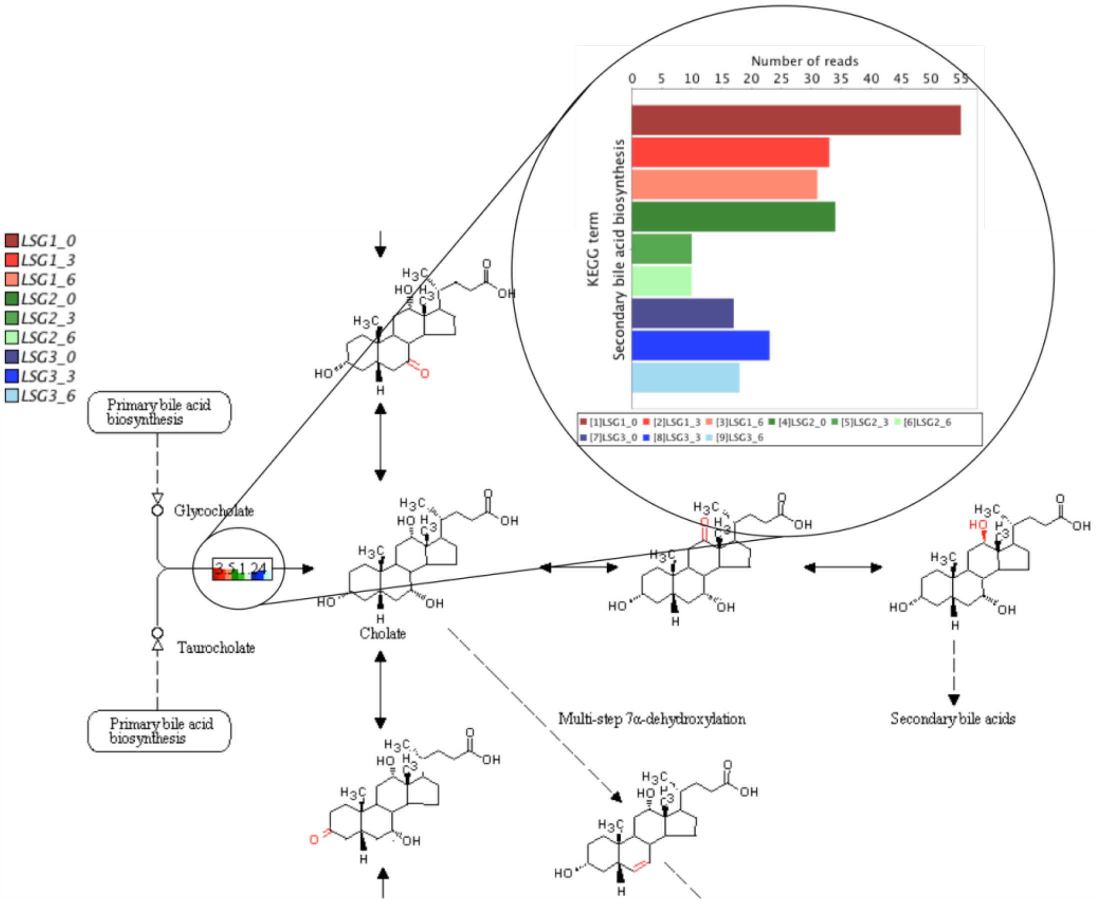

SI-Figure 5 Correlation and cluster analysis of fecal bile acids with intestinal microbes, which changed following laparoscopic sleeve gastrectomy. *Stars indicate significant correlations (* p<0.05, ** p<0.01). LCA, DCA: Secondary bile acids.*

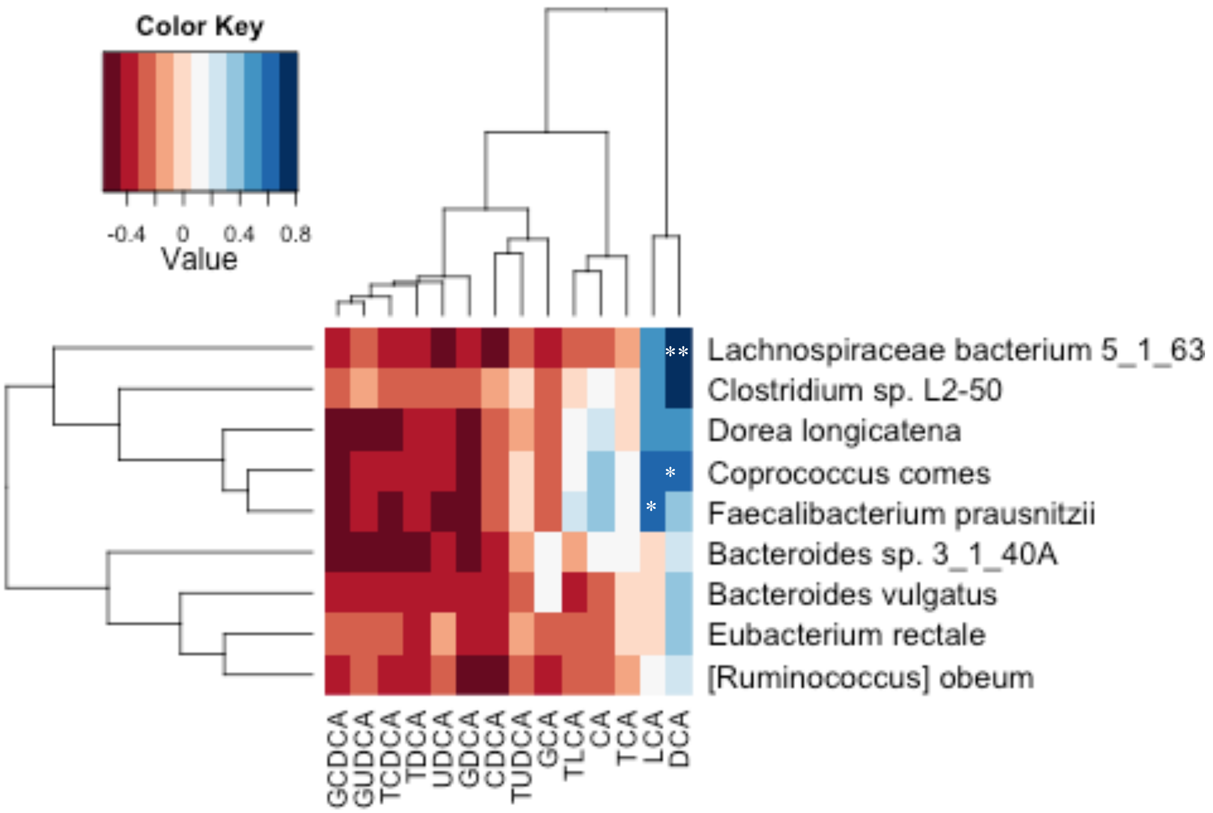

Supporting information 2: Tables

SI-Table 1: Alterations of the intestinal microbiota on different taxonomic levels following a) laparoscopic sleeve gastrectomy and b) Very low calorie diet-induced weight loss in obese subjects

Taxonomic level	*Bacteria category*	*Predictive coefficient[a]*	*P value[b]*	*Reads (normalized) Month 0*	*Reads (normalized) Month 3*	*Reads (normalized) Month 6*
a) Laparoscopic sleeve gastrectomy (LSG)						
Phylum	*Bacteroidetes/Chlorobi group*	0.8478	0.1667	77128.3 ± 5700.4	85069 ± 2011.1	86482.7 ± 2382.6
	Firmicutes	-0.8501	0.0952	15858.0 ± 5434.9	8488.7 ± 2025.2	6784.7 ± 2027.3
Class	*Bacteroidia*	0.8679	0.1667	75474.0 ± 5478.8	83494.7 ± 2170.1	84630.3 ± 1998.5
	Clostridia	-0.8725	0.0476*	14014.0 ± 5215.9	6572.3 ± 1196.3	5333.0 ± 1415.7
Order	*Bacteroidales*	0.8679	0.1667	75474.0 ± 5478.8	83494.7 ± 2170.1	84630.3 ± 1998.5
	Clostridiales	-0.8721	0.0476*	13978.0 ± 5212.3	6669.0 ± 1196.1	5320.3 ± 1412.0
Family	*Bacteroidaceae*	0.8790	0.1667	64302.0 ± 3587.6	69971.0 ± 2504.2	70006.3 ± 1345.0
	Eubacteriaceae	-0.7842	0.1667	5075.7 ± 3959.9	259.0 ± 178.3	652.0 ± 412.8
	Ruminococcaceae	-0.0908	0.0476*	1386.3 ± 448.0	605.0 ± 322.7	539.7 ± 319.0
Genus	*Bacteroides*	-0.8790	0.1667	64302.0 ± 3587.6	69971.0 ± 2504.2	70006.3 ± 1345.0
	Eubacterium	-0.8784	0.1667	5072.3 ± 3957.9	257.7 ± 178.8	650.7 ± 412.3
	Coprococcus[§]	-0.8902	0.0238*	**397.0 ± 135.2**	**116.3 ± 107.3**	**154.7 ± 120.3**
	Dorea	-0.9061	0.0238*	180.0 ± 28.3	72.7 ± 66.9	72.3 ± 49.7
	Faecalibacterium	-0.8053	0.2619	380.7 ± 48.7	163.3 ± 194.3	167.3 ± 196.7
	Ruminococcus	-0.7700	0.0476*	914.7 ± 506.3	357.7 ± 80.6	307.3 ± 140.7
Species	*Bacteroides sp.3_1_40A*	-0.9155	0.0238*	304.3 ± 59.7	142.3 ± 68.5	150.0 ± 88.8
	Bacteroides vulgatus	-0.7785	0.0952	4731.7 ± 2283.9	1913.7 ± 1788.5	1814.0 ± 1481.3
	Clostridium sp. L2-50[$]	-0.8967	0.0269*	**50.3 ± 10.4**	**18.3 ± 7.2**	**27.7 ± 7.4**
	Eubacterium rectale	-0.7927	0.1667	4370.0 ± 3501.9	103.7 ± 119.4	383.7 ± 486.8
	Ruminococcus obeum	-0.8239	0.0476*	143.0 ± 81.1	53.3 ± 36.5	33.0 ± 8.5
	Coprococcus comes	-0.8842	0.0952	269.7 ± 61.0	78.3 ± 79.1	124.3 ±111.2
	Dorea longicatena	-0.9053	0.0238*	132.0 ± 17.1	43.7 ± 38.5	53.0 ± 42.6
	Lachnospiraceae bacterium 5_1_63FAA	-0.8476	0.1667	65.3 ± 40.5	14.3 ± 11.4	11.3 ± 4.7
	Faecalibacterium prausnitzii	-0.8053	0.2619	380.7 ± 48.7	163.3 ± 194.3	167.3 ± 196.7

Taxonomic level	Bacteria category	Predictive coefficient[a]	P value[b]	Reads (normalized) Month 0	Reads (normalized) Month 3	Reads (normalized) Month 6
b) Very low calorie diet (VLCD)						
Phylum	***Fusobacteria***[§]	0.8155	0.0476*	**39.0 ± 46.2**	**97.7 ± 20.5**	**107.0 ± 16.7**
Class	***Bacilli***[§]	0.8429	0.0238*	**609.3 ± 319.8**	**1519.0 ± 375.4**	**2400.7 ± 1150.7**
Order	***Lactobacillales***[§]	0.8479	0.0238*	**485.7 ± 210.1**	**1257.3 ± 385.7**	**1966.7 ± 954.6**
	Fusobacterales[§]	0.8155	0.0476*	**39.0 ± 46.2**	**97.7 ± 20.5**	**107.0 ± 16.7**
Family	*Aerococcaceae*	0.7963	0.2619	46.0 ± 68.7	100.7 ± 10.5	183.3 ± 105.6
	Enterococcaceae[§]	0.7954	0.0238*	**85.7 ± 74.2**	**348.7 ± 162.4**	**482.7 ± 256.8**
	Peptococcaceae	0.7586	0.2619	27.3 ± 33.0	67.0 ± 15.1	90.0 ± 52.0
	Peptostreptococcaceae[§]	0.8301	0.0238*	**92.7 ± 94.0**	**263.3 ± 10.6**	**471.7 ± 174.8**
	Ruminococcaceae[§]	0.8842	0.0238*	**9498.7 ± 8154.5**	**28360.0 ± 7046.3**	**52656.3 ± 24157.4**
	UnclassifiedRum Clostridiales	0.9275	0.0238*	1262.0 ± 446.0	3158.0 ± 172.6	3179.3 ± 706.3
	Fusobacteriaceae[§]	0.8557	0.0238*	**31.0 ± 33.2**	**90.0 ± 14.1**	**95.3 ± 9.6**
Genus	***Enterococcus***[§]	0.7940	0.0238*	**85.7 ± 74.2**	**347.3 ± 163.7**	**482.7 ± 256.8**
	Lactobacillus[§]	0.7583	0.0476*	**27.3 ± 32.3**	**83.0 ± 19.9**	**204.0 ± 131.5**
	Oribacterium	0.7832	0.0238*	36.7 ± 36.5	131.3 ± 58.0	141.3 ± 45.7
	Ruminococcus	0.9321	0.0476*	6951.3 ± 6549.9	20517 ± 6500.0	34982.3 ± 8870.5
	Coprobacillus	0.8627	0.0238*	107.7 ± 98.7	711.0 ± 110.1	800.7 ± 251.9
	Holdemania	0.7672	0.0238*	48.7 ± 42.3	406.0 ± 154.9	357.7 ± 101.2
Species	*Enterococcus faecium*	0.8705	0.0238*	49.3 ± 45.0	215.3 ± 113.8	265.7 ± 69.8
	Clostridium saccharolyticum[§]	0.9435	0.0238*	**160.3 ± 49.0**	**554.7 ± 130.5**	**591.3 ± 56.0**
	Clostridium sp. D5	0.8852	0.0238*	79.0 ± 68.7	521.3 ± 233.8	594.3 ± 206.2
	Clostridium sp. M62/1	0.9416	0.0238*	348.3 ± 315.2	909.7 ± 160.6	1033.7 ± 232.1
	Clostridium symbiosum	0.8330	0.0238*	299.7 ± 147.7	1690.3 ± 798.0	1685.7 ± 327.8
	Eubacterium cellulosolvens[§]	0.7998	0.0952	36.0 ± 35.5	120.3 ± 80.1	224.0 ± 141.2
	Eubacterium limosum	0.9025	0.0238*	24.0 ± 25.6	105.0 ± 38.0	103.0 ± 28.1
	Blautia hydrogenotrophica	0.8775	0.0238*	96.7 ± 55.2	293.7 ± 30.6	294.7 ± 124.6
	Butyrivibrio fibrisolvens	0.8039	0.1667	35.0 ± 39.0	73.0 ± 38.0	131.7 ± 66.2
	Coprobacillus sp. 29_1	0.8558	0.0238*	94.0 ± 83.8	509.3 ± 113.2	663.7 ± 289.9

Shown are coefficients > 0.75 and < -0.75; [a] derived from OPLS-DA models (LSG: Q^2=0.337, R^2X=0.265, VLCD: Q^2=0.56, R^2X=0.216) with class discrimination pre (Month 0) to post (Month 3 and 6 combined) intervention; [b] Mann-Whitney-U Test comparing pre- to post-intervention; [§] (bold) significant changes (p=0.0278) for time course (Friedman test)
